# Genetic architecture of well-being: cumulative effect of serotonergic polymorphisms

**DOI:** 10.1093/scan/nsad039

**Published:** 2023-08-12

**Authors:** Yuhe Fan, Yuting Yang, Lele Shi, Wenping Zhao, Feng Kong, Pingyuan Gong

**Affiliations:** College of Life Science, Northwest University, Xi’an 710069, China; College of Life Science, Northwest University, Xi’an 710069, China; College of Life Science, Northwest University, Xi’an 710069, China; College of Life Science, Northwest University, Xi’an 710069, China; School of Psychology, Shaanxi Normal University, Xi'an 710062, China; College of Life Science, Northwest University, Xi’an 710069, China; College of Medicine, Northwest University, Xi’an 710069, China; Institute of Population and Health, Northwest University, Xi’an 710069, China; Key Laboratory of Resource Biology and Biotechnology in Western China, Ministry of Education, Northwest University, Xi’an 710069, China

**Keywords:** affective balance, global life satisfaction, psychological well-being, serotonin system, cumulative genetic score

## Abstract

Serotonin influences mental health and well-being. To understand the influences of genetic variations in serotonin pathway on well-being, we examined the effects of seven serotonergic polymorphisms on subjective well-being (i.e. affective balance and global life satisfaction) and psychological well-being (i.e. optimal psychological functions in the face of existential challenges) in a larger sample. Results indicated that the cumulative genetic score, but single genetic effects of serotonergic polymorphisms, was related to individual differences in well-being. Specifically, individuals with a greater cumulative genetic score, which is related to a low risk of depression, tended to exhibit high levels of subjective well-being and psychological well-being. These findings suggest that the overall serotoninergic genetic profile, rather than a specific genetic polymorphism, could greatly influence the individual differences in well-being.

## Introduction

Well-being (i.e. happiness) refers to individuals’ positive psychological state and a sense of personal growth and self-determination that people attain from life experiences (Diener *et al.*, 1998; Ryan and Deci, 2001). According to the telic theory of happiness (Diener, 2009), both satisfaction of needs and progress toward a goal can lead to happiness, but pursuit of unfulfilled goals causes unhappiness. The pursuit of happiness is a fundamental motivation underlying human actions. However, not all people have the same ability to create a good life. Research suggests that individual differences in well-being partly arise from biological factors, with an average heritability of 0.30–0.40 (Franz *et al.*, 2012; Vukasovic *et al.*, 2012).

There are two influential views on the aspects of well-being (Ryan and Deci, 2001; Keyes *et al.*, 2002). Subjective well-being refers to individuals’ overall appraisal and emotional experiences of life (Diener *et al.*, 1998; Ryan and Deci, 2001), reflecting the extent to which people believe they are doing well in life (Diener *et al.*, 1998; Keyes *et al.*, 2002). Psychological well-being refers to optimal psychological functions, such as the meaning of life and personal growth, which addresses an individual’s potential to live a meaningful life (Ryan and Deci, 2001; Keyes *et al.*, 2002). Empirical evidence indicates that the two aspects of well-being are strongly related, but strikingly distinctive (Chen *et al.*, 2013). Inspired by the robust genetic predispositions of well-being (Franz *et al.*, 2012; Nes and Roysamb, 2017), we investigated whether subjective well-being and psychological well-being share some of genetic foundations.

There have been many efforts to identify specific genetic factors of well-being (De Neve, 2011; De Neve *et al.*, 2012; Gartner *et al.*, 2018; Ohtsubo *et al.*, 2022). For example, an early study indicated that the 5-HTTLPR was related to general life satisfaction (De Neve, 2011), which was not replicated by their follow work (De Neve *et al.*, 2012), and a recent study indicated that this polymorphism was associated with specific life satisfaction, such as housing and family life satisfaction (Lachmann *et al.*, 2021). Additionally, the relationship between the 5-HTTLPR and well-being is moderated by demographic characteristics and life experiences. For instance, the S allele of 5-HTTLPR was related to lower life satisfaction in young people with early life stress, whereas the L allele of this polymorphism was associated with lower well-being in old people with early life stress (Gartner *et al.*, 2018). Generally, scientific communities have taken great efforts to investigate the effects of a single serotonergic variant on well-being. However, considering that the genetic foundations of well-being consist of multiple genes (Franz *et al.*, 2012; Vukasovic *et al.*, 2012), we estimated the extent to which the cumulative genetic effect of serotonergic pathway could influence well-being.

Several metabolic processes regulate serotonergic signaling. For example, serotonin biosynthesis is determined by the activity of tryptophan hydroxylase, and serotonergic synapses are modulated by serotonin transporter and serotonin receptors (Bockaert *et al.*, 2021; Pourhamzeh *et al.*, 2021). Research has shown that rs4570625 and rs7305115 of tryptophan hydroxylase 2 gene (*TPH2*) are associated with the enzyme activity of tryptophan hydroxylase 2 (Lim *et al.*, 2007; Chen *et al.*, 2008). The rs6295 (i.e. −1019C/G) of serotonin receptor 1A gene (*5-HT1A*) and the rs6311 (−1438A/G) and rs6313 (T102C) of serotonin receptor 2A gene (*5-HT2A*) are associated with serotonin receptors expressions (Polesskaya and Sokolov, 2002; Le Francois *et al.*, 2008). The 5-HTTLPR and 5-HTTVNTR of serotonin transporter gene (*5-HTT*) are related to the expression of serotonin transporter (Greenberg *et al.*, 1999). Additionally, the rs6311 of *5-HT2A* was found to be associated with the experiences of happiness during empathy or sharing of happiness (Matsunaga *et al.*, 2017, 2022). Inspired by the different roles of serotonergic genes, we examined whether the multiple serotonergic polymorphisms are differentially associated with well-being.

Candidate gene analysis has an advantage in exploring the associations between specific genes and well-being within existing neurobiological information framework (De Neve, 2011; De Neve *et al.*, 2012; Gartner *et al.*, 2018; Ohtsubo *et al.*, 2022), while genome-wide association analysis has an advantage in identifying new genetic factors of well-being at a genome-wide scale (Okbay *et al.*, 2016; Kim *et al.*, 2022). However, the effect of a single gene is relatively smaller (Okbay *et al.*, 2016; Chu *et al.*, 2020), and the analyses with sample sizes in the hundreds are underpowered to detect the genetic effect. To better understand the contributions of multiple genes to behavioral outcomes in relatively smaller samples, researchers have turned to use the approach of cumulative genetic score, in which similar behavioral effects of multiple polymorphisms are aggregated into a single score (Disner *et al.*, 2014; Pearson *et al.*, 2014). Therefore, due to the advantage of cumulative genetic score in estimating genetic architecture of complex behaviors, we quantified the effects of serotonergic polymorphisms on well-being with the approach of cumulative genetic score.

## Methods

### Participants

A priori power analysis suggests that a sample size of 787 participants would be needed to achieve a power of 80% (1−*β* = 0.80) at a significance level of 0.05 (two-tailed *α* = 0.05) when expected regression coefficient is close to 0.01 (Faul *et al.*, 2007). Considering the uncertainty in expected effect size, we set the sample size at 125% of estimation and invited 994 college students (681 females, mean age = 22.79 ± 1.88 years), suggesting the possibility of 88.2% for detecting a statistical significance. The participants were Han Chinese, with no selection or rejection criteria. This study was approved by the ethics committee of College of Life science, Northwest University, China.

### Well-being assessment

Subjective well-being includes the aspects of affect balance and global life satisfaction (Diener *et al.*, 1998; Keyes *et al.*, 2002). Affect balance (i.e. the balance of positive to negative emotions that an individual experienced) is typically scored by subtracting the negative affect score from the positive affect score. In this study, we measured the positive affect and negative affect with the Chinese version of the Scale of Positive and Negative Experience (Diener *et al.*, 2010). This scale consists of 12 items, and participants rated how often they experienced each affective state in the past 4 weeks (‘1’ = never, ‘5’ = almost always). The scores on positive and negative affect were computed by averaging the responses to positive and negative affect items, respectively. Cronbach’s alpha was 0.90.

Global life satisfaction was measured with the Chinese version of Satisfaction with Life Scale (Diener *et al.*, 1985). This 5-itemed scale assesses overall cognitive evaluation on life satisfaction by asking participants to rate how much they agree with the statements about their life satisfaction (‘1’ = strongly disagree and ‘7’ = strongly agree). The score for global life satisfaction was calculated by averaging the responses to five items. Cronbach’s alpha for the Satisfaction with Life Scale was 0.87.

Psychological well-being was assessed with the Chinese version of the Flourishing Scale (Diener *et al.*, 2010). This 8-itemed scale measures an individual’s overall sense of success in terms of human functions and needs including positive relationships, self-esteem, meaning and purpose of life and optimism. For each item (e.g. ‘*I lead a purposeful and meaningful life*’), participants indicated their feelings on a 7-point Likert scale (‘0’ = strongly disagree, ‘5’ = strongly agree). The score was calculated by averaging the responses to eight items and the Cronbach’s *α* was 0.89.

The paper-and-pencil tests began with a subjective well-being assessment, followed by a psychological well-being assessment in groups of 20–25 participants. To ensure common method variance (i.e. the tendency for participants to respond similarly to different measures due to a shared method of assessment) did not influence the results, we conducted a factor analysis. The results indicated only 37.26% of the variance was captured by the first un-rotated factor, which suggests that common method variance was not a significant issue in this study.

### Genotyping

We genotyped seven serotonergic polymorphisms by collecting 3–5 hair follicle cells from each participant to extract genomic DNA (de Lamballerie *et al.*, 1994). The rs4570625 and rs7305115 of *TPH2* are associated with serotonin biosynthesis. The rs6295 of *5-HT1A* is related to the firing rate of serotonergic neurons and a feedback on serotonin releasing (Sprouse and Aghajanian, 1987). The rs6311 and rs6313 of *5-HT2A* are associated with serotonergic excitatory at post-synaptic membrane (Barnes and Sharp, 1999). The 5-HTTLPR and 5-HTTVNTR of *5-HTT* are related to taking back of serotonin into presynaptic terminals (Greenberg *et al.*, 1999). These polymorphisms were amplified with polymerase chain reaction (PCR) method. The PCR system included 2.50 µl of 2 × reaction MIX (Golden Easy PCR System, TIANGEN), 0.50 µl of DNA Template, 1.50 µl of ddH_2_O, 0.25 µl (25 pmol/µl) upstream primer and 0.25 µl (25 pmol/µl) downstream primer. We genotyped the polymorphisms with the methods of PCR combined with restriction fragment length polymorphism or PCR with polyacrylamide gel electrophoresis (table S1 in Supplemental materials). To ensure the reliability of genotyping results, we randomly selected six samples to commercially sequence and 3% samples to re-genotype. The results were agreement with the first genotyping results, suggesting a high accuracy of our genotyping. Of note, the rs6295, rs6313 and 5-HTTLPR deviated from the Hardy–Weinberg equilibrium (HWE).

### Statistical analysis

The associations between single polymorphisms and well-being were examined with empirical multiple linear regression and Bayesian multiple linear regression. In empirical liner regression, genotype and standardized *Z* score of well-being were included as an independent variable and an outcome, respectively. In Bayesian linear regression, genotypes were used as independent variables in an alternative model to compare with a null model without any predictors, with Bayesian factor (BF_10_) indicating the evidence in favor of alternative hypotheses over the null hypotheses. Given that there were three outcomes (i.e. affective balance, global life satisfaction, and psychological well-being) and seven independent variables (i.e. seven polymorphisms), we set the significance level at *P *< 0.0025 (0.05/21) for the effect of single polymorphisms after Bonferroni-corrections. In the following analyses, the associations were accepted only when both statistical approaches produce significant estimations.

We also used empirical linear regression and Bayesian linear regression to examine the relationships between cumulative genetic score and the aspects of well-being. Due to no available criteria for encoding the genotypes and weighting the genetic effects, we treated the risk alleles of depression as ‘0’ and protective alleles as ‘1’ according to the results of meta-analyses (Figures S1–S7 in Supplemental materials), and weighted the genetic effect with standardized regression coefficient of each polymorphism impacting on well-being. Based on these procedures, we created a cumulative genetic score by summarizing the product of standardized regression coefficients of each polymorphism on well-being with the number of protective alleles. For analysis on the effect of cumulative genetic score, the significance level was set at *P* < 0.016 (0.05/3) because three outcomes and one independent variable (i.e. the cumulative genetic score) were involved.

## Results

### Associations between single polymorphisms and well-being

Collinearity diagnostics indicated that there was no significant collinearity among the polymorphisms when they were used as independent variables in predicting well-being (the variance inflation factor = 1.004–2.170). Empirical multiple linear regression indicated that all the polymorphisms were not significantly associated with affective balance (*P*s ≥ 0.146; Table 1), and Bayesian multiple linear regression also provide strong/moderate evidence for the lack of the associations, with BF_10_ ranging from 0.073 to 0.534. Similarly, the polymorphisms were not significantly associated with psychological well-being (*P*s ≥ 0.060; Table 1), and Bayesian multiple linear regression also provided strong/moderate evidence for lack of the associations (BF_10_ = 0.073–0.340). For global life satisfaction, empirical multiple linear regression showed that the rs6313 of *HTR2A* gene and rs4570625 of *TPH2* were significantly associated with this aspect of well-being (rs6313: standardized *β *= 0.108, *t *= 2.328, *P* = 0.020 and rs4570625: standardized *β *= 0.078, *t *= 2.003, *P* = 0.045). However, such statistical significances disappeared after Bonferroni-correction (corrected *P *= 0.420 for rs6313 and corrected *P *= 0.945 for rs4570625), and the Bayesian multiple linear regression analysis only provided weak evidence for the existence of the associations (BF_10_ = 1.120–2.164). Additionally, there was no significant interaction between the single nucleotide solymorphisms within any of the genes in the aspects of well-being after Bonferroni-correction (table S5 in Supplemental materials).

**Table 1.** The relationships between the single polymorphisms and well-being

	Genotype encoding			Affective balance				Global life satisfaction				Psychological well-being			
Polymorphism	0	1	2	*β*	*t*	*P*	BF_10_	*β*	*t*	*P*	BF_10_	*β*	*t*	*P*	BF_10_
rs6295	GG/CG	CC		0.028	0.881	0.378	0.094	−0.017	−0.548	0.054	0.089	0.058	1.828	0.068	0.340
rs6311	AA	AG	GG	−0.001	−0.030	0.976	0.221	−0.033	−0.713	0.476	0.214	0.008	0.167	0.867	0.077
rs6313	TT	TC	CC	0.068	1.455	0.146	0.534	0.108	2.328	0.020	2.164	0.004	0.095	0.924	0.074
rs4570625	GG	GT	TT	0.048	1.234	0.218	0.415	0.078	2.003	0.045	1.120	−0.029	−0.745	0.456	0.075
rs7305115	GG	AG	AA	0.025	0.637	0.524	0.257	0.001	0.016	0.988	0.171	0.073	1.880	0.060	0.328
5-HTTVNTR	LS/SS	LL		−0.012	−0.387	0.699	0.077	0.000	−0.009	0.993	0.071	−0.042	−1.334	0.183	0.185
5-HTTLPR	SS	LS/LL		−0.009	−0.292	0.770	0.073	0.036	1.127	0.260	0.131	0.006	0.175	0.861	0.073

Note: *β* refers to standardized regression coefficient.

### Relationship between the cumulative genetic effect across genes and well-being

**Fig. 1.**
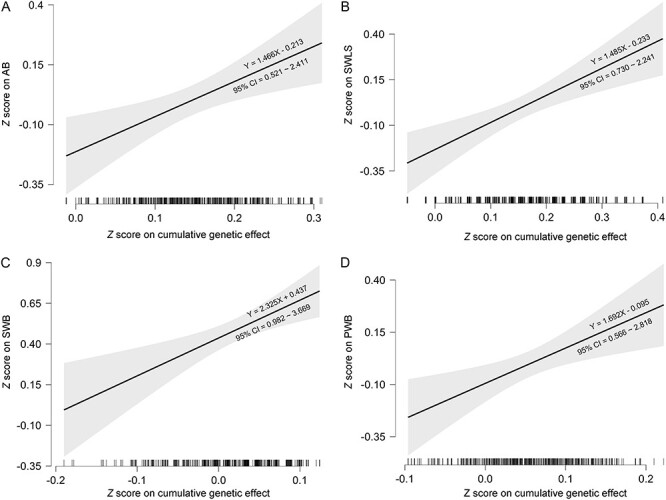
The scores on (A) affective balance (AB), (B) global life satisfaction (SWLS), (C) subjective well-being (SWB), and (D) psychological well-being (PWB) increase as a function of greater cumulative genetic effect of serotonergic polymorphisms (regression coefficients equation is unstandardized *β*).

Cumulative genetic score was created by summing up the products of standardized regression coefficient of each polymorphism on well-being and the number of protective alleles (table S6–S8 in Supplemental materials). Empirical liner regression indicated that the cumulative genetic score of serotonergic polymorphisms was significantly associated with affective balance (*β *= 0.096, *t *= 3.045, *P *= 0.002, corrected *P* = 0.006; Figure 1A), and Bayesian linear regression also provided moderate evidence for this association (BF_10_ = 6.704). Similarly, the cumulative genetic score was significantly associated with global life satisfaction (*β *= 0.122, *t *= 3.857, *P *< 0.001, corrected *P *= 0.003; Figure 1B), and Bayesian linear regression provided extreme evidence for the association (BF_10_ = 103.203). After combining affective balance with global life satisfaction as a global index of subjective well-being, the cumulative genetic score was also significantly associated with this index (*β *= 0.107, *t *= 3.396, *P* = 0.001, corrected *P *= 0.003; Figure 1C), and BF provided strong evidence for this association (BF_10_ = 20.223). In addition, the cumulative genetic score could predict psychological well-being (*β *= 0.093, *t *= 2.949, *P* = 0.003, corrected *P *= 0.009; Figure 1D), and Bayesian linear regression provided moderate evidence for this association (BF_10_ = 5.060).

To examine whether the cumulative genetic score underpinned the shared or unique variances of subjective well-being and psychological well-being, we included an aspect of well-being as an outcome and the other as a covariate in empirical liner regression. The results indicated that the significant relationship between the cumulative genetic score and psychological well-being survived when controlling for subjective well-being (*β *= 0.094, *t *= 2.970, *P* = 0.003), and the same was true for the relationship between the cumulative genetic score and subjective well-being when controlling for psychological well-being (*β *= 0.108, *t *= 3.418, *P *= 0.001).

## Discussion

Previous findings have shown that the 5-HTTLPR is associated with global life satisfaction or subjective well-being (De Neve *et al.*, 2012; Lachmann *et al.*, 2021; Ohtsubo *et al.*, 2022), while we did not found any significant associations. The lack of statistical significance may be due to the small effect size of each polymorphism and the potential moderations of other factors. Firstly, well-being is influenced by multiple genes with smaller effects (Okbay *et al.*, 2016; Chu *et al.*, 2020). Secondly, the effect of a single polymorphism may be modulated by other genetic factors (Van der Auwera *et al.*, 2014). Considering the lack of statistical significance in our relatively larger sample, we think it is not an effective approach to explain the individual differences in well-being by detecting the effect of a single gene.

This study found that individuals with a greater cumulative genetic score of serotonergic polymorphisms, indexing a predisposition of lower risk of depression, tend to exhibit higher level of affective balance. These findings demonstrate the importance of the cumulative genetic effect for affective balance. Affective balance is an indicator of emotion regulation (Lo *et al.*, 2021). Given that emotion regulation is modulated by the polymorphisms of 5-HTTLPR in *5-HTT* and the −1019C/G in *5-HT1A* (Canli and Lesch, 2007; Le Francois *et al.*, 2008; Barzman *et al.*, 2015), the effects of serotonergic polymorphisms on emotion regulation maybe contribute to the relationship between the cumulative genetic effect and affective balance. Additionally, these findings implicate the importance of this cumulative genetic effect for default brain settings because of the roles of affective balance in the default brain settings of activating positive affect and inactivating negative affect (Grinde, 2016). Moreover, adjusting affective balance within an optimal range has been shown to be an effective strategy for reducing depressive symptoms (Garamoni *et al.*, 1992). These findings maybe provide new insights into the relationships between serotonergic polymorphisms and depression (Le Francois *et al.*, 2008; Barzman *et al.*, 2015).

This study shows that a greater cumulative genetic score of serotonergic polymorphisms is associated with higher global life satisfaction. Global life satisfaction is a cognitive evaluation of individuals’ overall life satisfaction (Diener, 1984; Diener *et al.*, 1998). During this process, people examine their life, weigh the good against the bad, and arrive at a judgment of overall life satisfaction. If their judgment exceeds a certain standard or is better than others, they will be satisfied with their life (Diener, 1984). Considering that the evaluation on global life satisfaction is influenced by cognitive styles of self-evaluation and social comparison (Mehlsen *et al.*, 2005; Carrieri, 2012; Jiang *et al.*, 2014; Odaci *et al.*, 2019), and that serotonergic polymorphisms are related to cognitive styles, such as attribution style and cognitive flexibility (Hayden *et al.*, 2008; Sheikh *et al.*, 2008; Szily *et al.*, 2008; Gong *et al.*, 2011), we suppose that the relationship between the cumulative genetic score and global life satisfaction may be mediated by the influences of these polymorphisms on cognitive styles.

In contrast to previous studies that focused on the relationships between candidate genes and subjective well-being (Gartner *et al.*, 2018; Lachmann *et al.*, 2021; Ohtsubo *et al.*, 2022), this study extends the research field on the relationship between genetic effects and psychological well-being, with findings that individuals with a higher cumulative genetic score tend to live a meaningful life and exhibit higher levels of personal growth, self-esteem, and optimism. Considering that personalities such as extraversion and optimism are strong predictors of psychological well-being (Garaigordobil *et al.*, 2009; Butkovic *et al.*, 2012), and that there are known relationships between these personalities and serotonergic polymorphisms (Samochowiec *et al.*, 2001; Kazantseva *et al.*, 2008; Cam *et al.*, 2010; Lehto *et al.*, 2015), we think that the relationship between the cumulative genetic score and psychological well-being may be mediated by the influence of serotonergic polymorphisms on personalities.

Subjective well-being and psychological well-being load separately but related prosperities (Keyes *et al.*, 2002; Joshanloo, 2016). As the cumulative genetic effect is related to the both subjective well-being and psychological well-being, we tested whether it underpins the shared variances of two aspects of well-being. It is interesting to note that the relationship between the cumulative genetic score and subjective well-being is independent of psychological well-being, and vice versa. This suggests that the cumulative genetic effect may regulate unique, rather than shared, variances in both aspects of well-being through different pathways. For instance, the cumulative genetic effect may influence psychological well-being through sunny dispositions and sanguine outlook (Garaigordobil *et al.*, 2009; Butkovic *et al.*, 2012), regulate affective balance through emotion regulation (Nyklicek, 2011; Khosla, 2012), and impact global life satisfaction through cognitive styles (Mehlsen *et al.*, 2005; Carrieri, 2012; Jiang *et al.*, 2014; Odaci *et al.*, 2019). Overall, these findings highlight the importance of the cumulative genetic effect on different aspects of well-being. However, the bio-psychological mechanism of the cumulative genetic effect influencing on well-being should be investigated.

This study suggests that the cumulative genetic score, rather than the effect of a single polymorphism, may be a more effective predictor of well-being, which further suggests the advantage of this approach in improving statistical power and generating genetic variances when compared with examining a polymorphism in isolation (Disner *et al.*, 2014; Pearson *et al.*, 2014). We suppose that there are three reasons for the greater power of cumulative genetic score to capture the genetic effects. One possibility is that the cumulative genetic score could capture synergistic effects across multiple genes because the genetic foundations of well-being consist of multiple genes (Franz *et al.*, 2012; Vukasovic *et al.*, 2012). Another possibility is that this analysis approach takes into account the genetic background of serotonergic pathway and can explain more genetic variances, where the similar behavioral effects of multiple polymorphisms on well-being are modeled concurrently as a stronger genetic predictor (Pearson *et al.*, 2014). Thirdly, we made two improvements in generating cumulative genetic score when compared with previous studies (Disner *et al.*, 2014; Pearson *et al.*, 2014). Specifically, we determined the directions of putative genetic effects according to the protective effects of these polymorphisms on depression, given that there is a negative correlation between well-being and depression (Garamoni *et al.*, 1991). In contrast to simply summing up the number of risk alleles across polymorphisms (Disner *et al.*, 2014; Pearson *et al.*, 2014), we improved the accuracy of cumulative genetic score by weighting the product of the standardized regression coefficient for each polymorphism and the number of protective alleles.

Several limitations should be mentioned. Firstly, current measures on well-being were based on Western conceptions of well-being. Due to cultural differences in well-being (Joshanloo, 2014), the assessments may not perfectly gauge Chinese well-being. Secondly, this study was conducted among full-time college students. The demographic homogeneity reduced the generalization of our findings. Thirdly, mental health disorders influence how an individual responds to questions pertaining to well-being. If a participant suffers from psychiatric diseases or is taking antipsychotic medicines, her/his responses to questionnaires may be impacted. To guarantee accurate results, it would be advisable to exclude such individuals or statistically account for these variables. Fourthly, this study was based on a cross-sectional design. Longitudinal studies that follow individuals over time would be more informative in understanding how cumulative genetic score may influence the changes in well-being. Finally, rs6295, rs6313 and 5-HTTLPR deviated from the HWE, which may suggest a population stratification or selection bias. Due to the deviations may bias the estimations of genetic effects, these genetic variants we should be excluded in the analysis. However, considering the critical roles of these variants in serotonergic metabolism and signaling, especially in generating cumulative genetic score, we included these variants in analysis. Therefore, the findings should be examined in a more general population.

## Conclusion

This study demonstrates that the cumulative genetic effect of serotonergic polymorphisms can significantly predict both subjective well-being and psychological well-being, suggesting the polygenic genetic architecture of well-being.

## References

[R1] Barnes N.M. , Sharp T. (1999). A review of central 5-HT receptors and their function. *Neuropharmacology*, 38(8), 1083–152. 10462127 10.1016/s0028-3908(99)00010-6

[R2] Barzman D. , Geise C., Lin P.I. (2015). Review of the genetic basis of emotion dysregulation in children and adolescents. *World Journal of Psychiatry*, 5(1), 112–7. 25815260 10.5498/wjp.v5.i1.112 PMC4369540

[R3] Bockaert J. , Becamel C., Chaumont-Dubel S., Claeysen S., Vandermoere F., Marin P. (2021). Novel and atypical pathways for serotonin signaling. *Faculty Reviews*, 10(52), 1–14. 34195691 10.12703/r/10-52 PMC8204760

[R4] Butkovic A. , Brkovic I., Bratko D. (2012). Predicting well-being from personality in adolescents and older adults. *Journal of Happiness Studies*, 13(3), 455–67.

[R5] Cam F.S. , Colakoglu M., Tok S., Tok I., Kutlu N., Berdeli A. (2010). Personality traits and DRD4, DAT1, 5-HT2A gene polymorphisms in risky and non risky sports participation. *Turkiye Klinikleri Tip Bilimleri Dergisi*, 30(5), 1459–64.

[R6] Canli T. , Lesch K.P. (2007). Long story short: the serotonin transporter in emotion regulation and social cognition. *Nature Neuroscience*, 10(9), 1103–9. 17726476 10.1038/nn1964

[R7] Carrieri V. (2012). Social comparison and subjective well-being: does the health of other matter?.*Bulletin of Economic Research*, 64(1), 31–55. 22299192 10.1111/j.1467-8586.2011.00393.x

[R8] Chen F.F. , Jing Y.M., Hayes A., Lee J.M. (2013). Two concepts or two approaches? A bifactor analysis of psychological and subjective well-being. *Journal of Happiness Studies*, 14(3), 1033–68.

[R9] Chen G.L. , Vallender E.J., Miller G.M. (2008). Functional characterization of the human TPH2 5′ regulatory region: untranslated region and polymorphisms modulate gene expression in vitro. *Human Genetics*, 122(6), 645–57. 17972101 10.1007/s00439-007-0443-y PMC2734478

[R10] Chu X.M. , Liu L., Wen Y., et al. (2020). A genome-wide multiphenotypic association analysis identified common candidate genes for subjective well-being, depressive symptoms and neuroticism. *Journal of Psychiatric Research*, 124, 22–8. 32109668 10.1016/j.jpsychires.2020.02.012

[R0010a] de Lamballerie X. , Chapel F., Vignoli C., Zandotti C. (1994). Improved current methods for amplification of DNA from routinely processed liver tissue by PCR. *Journal of Clinical Pathology*, 47(5), 466–7. 8027403 10.1136/jcp.47.5.466 PMC502029

[R11] De Neve J.-E. (2011). Functional polymorphism (5-HTTLPR) in the serotonin transporter gene is associated with subjective well-being: evidence from a US nationally representative sample. *Journal of Human Genetics*, 56(6), 456–9. 21562513 10.1038/jhg.2011.39

[R12] De Neve J.-E. , Christakis N.A., Fowler J.H., Frey B.S. (2012). Genes, economics, and happiness. *Journal of Neuroscience, Psychology, and Economics*, 5(4), 193–211. 10.1037/a0030292 PMC3858957 24349601

[R13] Diener E. (1984). Subjective well-being. *Psychological Bulletin*, 95(3), 542–75. 6399758

[R14] Diener E. (2009). Subjective well-being. In: Diener, E., editor. *Science of Well-Being: The Collected Works of Ed Diener*. Vol. 37, Springer Netherlands: Springer, 11–58.

[R15] Diener E. , Emmons R.A., Larsen R.J., Griffin S. (1985). The satisfaction with life scale. *Journal of Personality Assessment*, 49(1), 71–5. 16367493 10.1207/s15327752jpa4901_13

[R16] Diener E. , Sapyta J.J., Suh E. (1998). Subjective well-being is essential to well-being. *Psychological Inquiry*, 9(1), 33–7.

[R17] Diener E. , Wirtz D., Tov W., et al. (2010). New well-being measures: short scales to assess flourishing and positive and negative feelings. *Social Indicators Research*, 97(2), 143–56.

[R18] Disner S.G. , McGeary J.E., Wells T.T., Ellis A.J., Beevers C.G. (2014). HTR1A, and HTR2A cumulative genetic score interacts with mood reactivity to predict mood-congruent gaze bias. *Cognitive, Affective & Behavioral Neuroscience*, 14(4), 5–HTTLPR. 10.3758/s13415-014-0267-x PMC4169358 24643765

[R19] Faul F. , Erdfelder E., Lang A.-G., Buchner A. (2007). G*Power 3: a flexible statistical power analysis program for the social, behavioral, and biomedical sciences. *Behavior Research Methods*, 39(2), 175–91. 17695343 10.3758/bf03193146

[R20] Franz C.E. , Panizzon M.S., Eaves L.J., et al. (2012). Genetic and environmental multidimensionality of well- and ill-being in middle aged twin men. *Behavior Genetics*, 42(4), 579–91. 22484556 10.1007/s10519-012-9538-x PMC4056448

[R21] Garaigordobil M. , Aliri J., Fontaneda I. (2009). Subjective psychological well-being: gender differences, relations with personality dimensions and predictor variables. *Behavioral Psychology-Psicologia Conductual*, 17(3), 543–59.

[R22] Garamoni G.L. , Reynolds C.F., Thase M.E., Frank E., Berman S.R., Fasiczka A.L. (1991). The balance of positive and negative affects in major depression: a further test of the states of mind model. *Psychiatry Research*, 39(2), 99–108. 1798819 10.1016/0165-1781(91)90079-5

[R23] Garamoni G.L. , Reynolds C.F., Thase M.E., Frank E., Fasiczka A.L. (1992). Shifts in affective balance during cognitive therapy of major depression. *Journal of Consulting and Clinical Psychology*, 60(2), 260–6. 1592956 10.1037//0022-006x.60.2.260

[R24] Gartner M. , Grimm S., Aust S., Fan Y., von Scheve C., Bajbouj M. (2018). The interplay of genetic and environmental factors in shaping well-being across the lifespan: evidence from the serotonin transporter gene. *Aging & Mental Health*, 22(9), 1216–22. 28685605 10.1080/13607863.2017.1348467

[R25] Gong P.Y. , Zhang F.C., Ge W.H., et al. (2011). Association analysis of TPH2, 5-HT2A, and 5-HT6 with executive function in a young Chinese Han population. *Journal of Neurogenetics*, 25(1-2), 27–34. 21457069 10.3109/01677063.2011.569804

[R26] Greenberg B.D. , Tolliver T.J., Huang S.J., Li Q., Bengel D., Murphy D.L. (1999). Genetic variation in the serotonin transporter promoter region affects serotonin uptake in human blood platelets. *American Journal of Medical Genetics*, 88(1), 83–7. 10050973

[R27] Grinde B. (2016). Why negative feelings are important when assessing well-being. *Journal of Happiness Studies*, 17(4), 1741–52.

[R28] Hayden E.P. , Dougherty L.R., Maloney B., et al. (2008). Early-emerging cognitive vulnerability to depression and the serotonin transporter promoter region polymorphism. *Journal of Affective Disorders*, 107(1–3), 227–30. 17804080 10.1016/j.jad.2007.07.028 PMC2692689

[R29] Jiang W.S. , Li F., Jiang H.P., et al. (2014). Core self-evaluations mediate the associations of dispositional optimism and life satisfaction. *Plos One*, 9(6), e97752. 10.1371/journal.pone.0097752 PMC4049581 24911367

[R30] Joshanloo M. (2014). Eastern conceptualizations of happiness: fundamental differences with western views. *Journal of Happiness Studies*, 15(2), 475–93.

[R31] Joshanloo M. (2016). Revisiting the empirical distinction between hedonic and eudaimonic aspects of well-being using exploratory structural equation modeling. *Journal of Happiness Studies*, 17(5), 2023–36.

[R32] Kazantseva A.V. , Gaysina D.A., Faskhutdinova G.G., Noskova T., Malykh S.B., Khusnutdinova E.K. (2008). Polymorphisms of the serotonin transporter gene (5-HTTLPR, A/G SNP in 5-HTTLPR, and STin2 VNTR) and their relation to personality traits in healthy individuals from Russia. *Psychiatric Genetics*, 18(4), 167–76. 18628678 10.1097/YPG.0b013e328304deb8

[R33] Keyes C.L.M. , Shmotkin D., Ryff C.D. (2002). Optimizing well-being: the empirical encounter of two traditions. *Journal of Personality and Social Psychology*, 82(6), 1007–22. 12051575

[R34] Khosla M. (2012). Emotion regulation and well-being. *Applied Research in Quality of Life*, 7(3), 323–5.

[R35] Kim S. , Kim K., Hwang M.Y., et al. (2022). Shared genetic architectures of subjective well-being in East Asian and European ancestry populations. *Nature Human Behaviour*, 6(7), 1014-+. 10.1038/s41562-022-01343-5 35589828

[R36] Lachmann B. , Doebler A., Sindermann C., et al. (2021). The molecular genetics of life satisfaction: extending findings from a recent genome-wide association study and examining the role of the serotonin transporter. *Journal of Happiness Studies*, 22(1), 305–22.

[R37] Le Francois B. , Czesak M., Steubl D., Albert P.R. (2008). Transcriptional regulation at a HTR1A polymorphism associated with mental illness. *Neuropharmacology*, 55(6), 977–85. 18639564 10.1016/j.neuropharm.2008.06.046

[R38] Lehto K. , Vaht M., Maestu J., Veidebaum T., Harro J. (2015). Effect of tryptophan hydroxylase-2 gene polymorphism G-703 T on personality in a population representative sample. *Progress in Neuro-Psychopharmacology & Biological Psychiatry*, 57, 31–5. 25455586 10.1016/j.pnpbp.2014.10.005

[R39] Lim J.E. , PinsonneaultJ., SadeeW., SaffenD. (2007). Tryptophan hydroxylase 2 (TPH2) haplotypes predict levels of TPH2 mRNA expression in human pons. *Molecular Psychiatry*, 12(5), 491–501.1745306310.1038/sj.mp.4001923

[R40] Lo S.L. , Riley H.O., Sturza J., et al. (2021). Cortisol in early childhood moderates the association between family routines and observed affective balance in children from low-income backgrounds. *Developmental Psychobiology*, 63(8), e22204. 10.1002/dev.22204 PMC12848807 34813102

[R41] Matsunaga M. , Kawamichi H., Umemura T., et al. (2017). Neural and genetic correlates of the social sharing of happiness. *Frontiers in Neuroscience*, 11, 718. 10.3389/fnins.2017.00718 PMC5742108 29311795

[R42] Matsunaga M. , Ohtsubo Y., Masuda T., Noguchi Y., Yamasue H., Ishii K. (2022). Serotonin receptor (HTR2A) gene polymorphism modulates social sharing of happiness in both American and Japanese adults. *Japanese Psychological Research*, 64(2), 181–92.

[R43] Mehlsen M. , Thomsen D.K., Viidik A., Olesen F., Zachariae R. (2005). Cognitive processes involved in the evaluation of life satisfaction: implications for well-being. *Aging & Mental Health*, 9(3), 281–90. 16019282 10.1080/13607860412331310236

[R44] Nes R.B. , Roysamb E. (2017). Happiness in behaviour genetics: an update on heritability and changeability. *Journal of Happiness Studies*, 18(5), 1533–52.

[R45] Nyklicek I. (2011). Mindfulness, emotion regulation, and well-being. In: Nyklíček, I., Vingerhoets, A., Zeelenberg, M., editors. *Emotion Regulation and Well-Being*. New York, NY: Springer. 101–18.

[R46] Odaci H. , Cikrikci O., Cikrikci N., Aydin F. (2019). An exploration of the associations among cognitive flexibility, attachment styles and life satisfaction. *International Journal of Happiness and Development*, 5(3), 242–56.

[R47] Ohtsubo Y. , Matsunaga M., Masuda T., Noguchi Y., Yamasue H., Ishii K. (2022). Test of the serotonin transporter gene × early life stress interaction effect on subjective well-being and loneliness among Japanese young adults. *Japanese Psychological Research*, 64(2), 193–204.

[R48] Okbay A. , Baselmans B.M.L., De Neve J.E., et al. (2016). Genetic variants associated with subjective well-being, depressive symptoms, and neuroticism identified through genome-wide analyses. *Nature Genetics*, 48(6), 624-+. 10.1038/ng.3552 PMC4884152 27089181

[R49] Pearson R. , McGeary J.E., Beevers C.G. (2014). Association between serotonin cumulative genetic score and the behavioral approach system (BAS): moderation by early life environment. *Personality and Individual Differences*, 70, 140–4. 25264393 10.1016/j.paid.2014.06.041 PMC4174309

[R50] Polesskaya O.O. , Sokolov B.P. (2002). Differential expression of the “C” and “T” alleles of the 5-HT2A receptor gene in the temporal cortex of normal individuals and schizophrenics. *Journal of Neuroscience Research*, 67(6), 812–22. 11891796 10.1002/jnr.10173

[R51] Pourhamzeh M. , Moravej F.G., Arabi M., et al. (2021). The roles of serotonin in neuropsychiatric disorders. *Cellular and Molecular Neurobiology*, 42(6), 1671–92. 33651238 10.1007/s10571-021-01064-9 PMC11421740

[R52] Ryan R.M. , Deci E.L. (2001). On happiness and human potentials: a review of research on hedonic and eudaimonic well-being. *Annual Review of Psychology*, 52, 141–66. 10.1146/annurev.psych.52.1.141 11148302

[R53] Samochowiec J. , Rybakowski F., Czerski P., et al. (2001). Polymorphisms in the dopamine, serotonin, and norepinephrine transporter genes and their relationship to temperamental dimensions measured by the temperament and character inventory in healthy volunteers. *Neuropsychobiology*, 43(4), 248–53. 11340364 10.1159/000054898

[R54] Sheikh H.I. , Hayden E.P., Singh S.M., et al. (2008). An examination of the association between the 5-HTT promoter region polymorphism and depressogenic attributional styles in childhood. *Personality and Individual Differences*, 45(5), 425–8. 19122845 10.1016/j.paid.2008.05.020 PMC2583465

[R55] Sprouse J.S. , Aghajanian G.K. (1987). Electrophysiological responses of serotoninergic dorsal raphe neurons to 5-HT1A and 5-HT1B agonists. *Synapse*, 1(1), 3–9. 3505364 10.1002/syn.890010103

[R56] Szily E. , Bowen J., Unoka Z., Simon L., Keri S. (2008). Emotion appraisal is modulated by the genetic polymorphism of the serotonin transporter. *Journal of Neural Transmission*, 115(6), 819–22. 18273536 10.1007/s00702-008-0029-4

[R57] Van der Auwera S. , Janowitz D., Schulz A., et al. (2014). Interaction among childhood trauma and functional polymorphisms in the serotonin pathway moderate the risk of depressive disorders. *European Archives of Psychiatry and Clinical Neuroscience*, 264, 45–54. 25214390 10.1007/s00406-014-0536-2

[R58] Vukasovic T. , Bratko D., Butkovic A. (2012). Genetic contribution to the individual differences in subjective well-being: a meta-analysis. *Drustvena Istrazivanja*, 21(1), 1–17.

